# Improved Food-Processing Techniques to Reduce Isoflavones in Soy-Based Foodstuffs

**DOI:** 10.3390/foods12071540

**Published:** 2023-04-05

**Authors:** Souad Bensaada, François Chabrier, Pascal Ginisty, Carine Ferrand, Gabriele Peruzzi, Marc Valat, Catherine Bennetau-Pelissero

**Affiliations:** 1 Campus Carreire, Pharmacy Faculty, Department Sciences and Technology, University of Bordeaux, 33076 Bordeaux, France; carine.ferrand@u-bordeaux.fr (C.F.); marc.valat@u-bordeaux.fr (M.V.); 2 ARNA (Nucleic Acids: Natural and Artificial Regulations), U1212 Inserm, UMR CNRS 5320, University of Bordeaux, 33076 Bordeaux, France; 3 Biopress, 47400 Tonneins, France; gabriele.peruzzi@biopress.fr; 4 Agrotec, Agropole, 47310 Estillac, France; f.chabrier@agrotec-france.com; 5 IFTS (Institute of Filtration and Separative Techniques), 47510 Foulayronnes, France; pascal.ginisty@ifts-sls.com; 6 BFP (Fruit Biology and Pathology), UMR CNRS 1332, INRA Bordeaux-Aquitaine, University of Bordeaux, 33140 Villenave-d’Ornon, France; 7 I2M (Mechanical and Engineering Institute), UMR CNRS 5295, University of Bordeaux, 33405 Talence, France; 8 Bordeaux Sciences Agro, 33175 Gradignan, France

**Keywords:** genistein, daidzein, consumer exposure, manufacturing process, soybean, isoflavone removal

## Abstract

Soy is a growing protein source; however, the isoflavones it contains are of concern, as they exhibit estrogenic activities whose toxicological limits might be exceeded. Reducing their concentrations to safe levels while preserving nutritional quality in soy foodstuffs is therefore a matter of public health. The main objective of this paper is to develop at pilot scale a process for isoflavones’ extraction from soybeans, and to show its feasibility and efficiency. The study was conducted by first optimizing the previously obtained laboratory treatment key factors. These data were then transposed to the pilot level. Finally, the process was adjusted to technical constraints which appeared at pilot scale: the mandatory use of drenching and the exploration of granulometry analysis. The involved steps were validated by monitoring the genistein and daidzein content variations through statistical analysis of the data of an ELISA and a Folin–Ciocalteu assay. Additionally, isoflavones’ recovery from treatment waters for their valorisation and the water cleaning by means of filtration, centrifugation and resin adsorption were carried out. The results showed that the most successful pilot treatment developed involved soybean dehulling, drenching, washing and drying and almost halved isoflavones while preserving the main nutritional characteristics. A combination of techniques led to almost complete recovery of isoflavones from process waters.

## 1. Introduction

Isoflavones (IFs) are polyphenols bearing both estrogenic and antiestrogenic traits, among other properties. It has been shown that they exhibit beneficial health effects, especially in people with an oestrogen deficiency, i.e., women in natural or artificial menopause [1,2]. Indeed, IFs at doses of 40 mg/day or more could prevent vasomotor symptoms associated with menopause. Some other meta-analysis suggested that phytoestrogens from soy could prevent bone loss in menopause via an estrogenic effect [3,4,5], and even more efficiently under aglycone forms [6].

However, alongside these potential beneficial effects, menstrual cycle impairments were observed in women exposed to IFs in doses of about 0.75 mg/kg bw (body weight)/day in aglycone forms [7,8]. More recently, IF intake at a level of 50 mg/day in women, i.e., about 0.8 mg/kg bw/day, was shown to be significantly correlated with luteal phase impairment and with the risk of nulliparity at 26 years old [9,10]. In addition, five clinical or population studies have so far reported the association of high soy IFs intake and their biomarkers in body fluids with a reduction in sperm count and quality [11,12,13,14,15]. When studies reported no effects on semen quality in men, it was at low doses [16] or for too short exposure times [17]. Overconsumption of soy has also been linked to several adverse clinical cases in women [18] and men [19]. In the former, on one hand, menstrual cycles were impaired and contraceptive pill treatments suffered from interference, and on the other hand, large uterus fibrosis and endometriosis features were observed. The effects regressed after soy arrest. In men, a secondary hypogonadism, which is the impairment of sexual behaviour and the feature of gynecomastia, was observed. The hypogonadism, characterised by dramatically reduced sex steroid concentrations in the blood, tended to return to normal levels after 3 months without soy. IFs have also been the centre of a great controversy regarding breast cancer, with several studies concluding in favour of a protective effect, mainly in Asian populations [20], while others reporting a proliferative impact [21]. It is though currently admitted, thanks to a better understanding of their mechanisms of action, that IFs have a dual effect: protective during the promotion phase (healthy cells), but proliferative on already declared oestrogen-dependent cancer cells. For instance, the activation of GPER, inducing cell proliferation, and the inhibition of protein tyrosine kinase, inducing cell apoptosis, were reported with low and high IF doses, respectively [22,23].

Nowadays, the main dietary source of IFs for humans is soy and derived soy by-products [24]. Modern western soy transformation processes sometimes expose consumers to high IF concentrations in food [25,26,27]. Considering the whole spectrum of effects recorded in humans, this potential dietary exposure may be considered a concern, and the precaution principle should be applied for people who exhibit a particular sensitivity to oestrogens, i.e., infants, children, pregnant women [20] and those with high risk of oestrogen-dependent breast or endometrial cancers. Moreover, hypothyroid patients taking medication, namely levothyroxine, should avoid soy IFs [28,29]. IFs should instead be kept for natural medication when proven useful. A perfect illustration of this observation can be found in the Chinese pharmacopeia, in which IFs are used for their medicinal properties only as extracted from Kudzu roots [30], with soy never being considered a health ingredient. This is likely because ancient recipes of Asian soy-based food included water soaking, cooking and simmering, a process that removes most IF content, thereby reducing any health impact.

Toxicological data have been available since 2008 on genistein (GEN), the most commonly studied phytoestrogen from soy. They were published by the US National Toxicology Program and dealt with the reprotoxicity [31] and carcinotoxicity [32] of this isoflavone. The no observable adverse effect level (NOAEL) could not be obtained from these studies, so a lowest observable adverse effect level (LOAEL) was alternatively proposed for both reproduction and carcinotoxic effects, which was 35 mg/kg bw/day in rats. Such a dose may help to determine a reference dose (RefD), i.e., a limit of exposure for humans. Additionally, as discussed by Lee et al. [26], even if endocrine disruptors can be more deleterious at low doses, due probably to the activation of different pathways, as mentioned above, there is no evidence of any adverse effects of low IF doses in vivo and in humans. Rather, it is doses over 20–40 mg/d (aglycone–glycosidic forms) that can exert physiological activities, either beneficial or deleterious, depending on the status of the consumer.

In this context, a previous work [27] has already demonstrated at the laboratory level the feasibility of a strong reduction in the IF content of soybeans by a specific water treatment. An important issue ensued, and may be phrased by the following question: how can Ifs’ removal from soy matter be carried out on an industrial scale without degrading its nutritional quality and generating a heavy additional hurdle either? The purpose of the present investigation is (1) to provide a positive and convincing answer to this question by means of a pilot-scale study that could then be scaled up, (2) to mitigate the environmental impact and supplementary costs through water and energy savings, and (3) to recover IFs from treatment water to feed the phytochemical industry. Indeed, as IFs are active compounds, they could and should be used for health applications in rigorously controlled supplementation. Therefore, collecting them in soy-based food factories would also reduce unwanted environmental exposure.

To do this, the approach utilised is as follows. (1) To determine the optimum experimental configuration of design factors those deemed relevant for IFs’ removal [27] at the laboratory were first optimized by monitoring the content variations of the main soy IFs, GEN and daidzein (DAI) [33]. The individual impacts of these factors were evidenced by IF concentration measurements, taking advantage of the enzyme-linked immuno-sorbent assay (ELISA)’s high sensitivity and specificity. (2) To achieve improved treatment at pilot scale, the transposed data of the optimized laboratory factors were then readjusted. This was accomplished while exploring the impacts of unexpected (drenching) and relevant additional (granulometry, determined by laser diffraction and sieve analysis) factors. The whole process also implemented the supplementary constraint of the final step of drying the treated soybeans. (3) On another side, as the ELISA requires a rather long implementation, a faster assay method, i.e., Folin–Ciocalteu, was tested, since it appeared better suited to routine usage in an industrial environment. (4) The nutritional quality control of soy matter was performed by Phytocontrol^®^ (Nimes, France) according to Dumas and Kjeldhal’s methods for proteins and gravimetry for lipids. (5) To study the feasibility and quality of water recycling as well as IFS recovery, centrifugation, filtration and resin adsorption were tested on treatment waters. Appropriate statistical analyses were performed, i.e., signal to noise ratios for laboratory study and a Mann–Whitney test for pilot-scale data.

The results presented here are discussed under the realm of the potential exposure limit RefD, as defined above. The pilot-scale investigation resulted in the highlighting of a successful improved treatment that significantly reduced IF concentrations in soy.

## 2. Materials and Methods

### 2.1. Materials

All soy matter (whole beans, dehulled beans, pounded dehulled beans, soy cake and textured proteins) were supplied by Biopress^®^ (Tonneins, France), except the dehulled beans for the laboratory-scale experiments, which were purchased in an organic store. The chemical reagents were acquired from MERK (Fontenay-sous-Bois, France) and Sigma-Aldrich (Saint Quentin Fallavier, France), unless otherwise stated. The samples and reagents’ aqueous solutions were made in ultrapure water obtained from an Elga Veolia^®^ (High Wycombe, UK) instrument.

### 2.2. Isoflavones Reduction at Laboratory Scale

Based on the conclusions of a previous work, the treatment of soy matter through water washing, the effectiveness of which depends upon five factors [27], was implemented. To adjust it, the latter were tested at five different levels, each completing a Taguchi L25 design of experiment [34,35]. For each attempt, 10 g of matter was added to the corresponding water volume needed to reach a soy matter:water volume ratio of 1:2, 1:2.5, 1:3, 1:4 or 1:5. The approximation that the soy matter density is 1 g/mL, regardless of the soy matrix, was made so that the sample volumes were easy to calculate. Treatments lasting 0, 5, 10, 20 and 30 min were carried out at room temperature (20–24 °C), 45 °C, 55 °C, 65 °C and 75 °C. The mixtures were stirred with either a magnetic or an orbital table stirrer at different speeds of 0, 50, 100 and 200 RPM (revolutions per minute). Each test was run in triplicate. The resulting soy samples were dried in a thermostatically controlled oven at 75 °C until a constant weight was reached, and then stored at −20 °C. The statistical analyses relied on the measurement of signal-to-noise ratios with XLSTAT software (Addinsoft_2020, XLSTAT, Paris, France).

### 2.3. Isoflavones Reduction at Pilot Scale

A batch of 6 kg of dehulled beans was washed using 24 L (volume ratio 1:4) of water at 20 °C for 10 min at 200 RPM in a 100 L tank. Another batch of the same weight was drenched before washing. For this purpose, the beans were spread out on a grid and exposed to 2 L of water sprayed through six nozzles for 13 s. The water used for both operations was kept close to 20 °C. As the batch which was both drenched and washed showed better results over the simply washed one, the corresponding assay was carried out three times.

The treated beans were then dried either by lick-drying (air temperature, velocity, and relative humidity equal to 55 °C, 2.25 m/s and 11%, respectively) or thick bed-drying (air temperature and velocity equal to 55 °C and 1.5 m/s, respectively).

To assess the achievement of satisfactory IF removal, the remaining IF concentrations in the treated dry soy matter were compared to values that have deleterious effects, as found in the literature.

### 2.4. Biochemical Analysis

#### 2.4.1. ELISA

##### Sample Preparation

In order to carry out the IF analyses by ELISA, the samples were first subjected to the treatment described below. Thereby, 1 g of mortar-crushed soy-based material was dispersed into 50 mL of water by two-step stirring at room temperature for 20 min, and then at 100 °C for 10 min. After cooling down to room temperature, 500 µL samples were collected in triplicate under stirring to insure homogeneity. Washing- and drenching-water samples were also treated as 500 µL triplicates. Two millilitres of acetate buffer (sodium acetate 0.1 M; EDTA 0.14 M; 100 UI/mL penicillin G (Sigma, P-3032); and 0.1 mg/mL streptomycin (Sigma, S-6501)) at pH 5 were added to each sample vial, together with 10 µL of β-glucuronidase aryl-sulfatase from *Helix pomatia* (Roche^®^, 10127698001, Mannheim, Germany) to allow the digestion of glycosylated IFs. The samples were then incubated overnight at 37 °C under shaking [36]. Afterwards, the extraction of aglycone compounds was performed: 3 mL of acidified ethyl-acetate (500 µL HCl 38% per L) was added; the vial vortexed for 30 s, centrifuged at 500 g (Jouan^TM^ CR3, Fisher Scientific, Illkirch, France) for 10 min at 4 °C and then stored at −22 °C to allow phase separation. The organic phase containing the IFs in aglycone forms was evaporated to dryness using a Speed-Vac (Thermo-Electron^TM^ Corporation, Fisher Scientific, Bordeaux, France). Afterwards, each sample was diluted in 0.5 mL of assay buffer, i.e., phosphate-buffered saline (PBS) 0.01 M, 0.9% NaCl, 0.2% Tween, 1% DMSO, pH 7.3, and sonicated when required. Samples were stored at −22 °C until they were processed for IF analysis.

To assess the digestion and extraction recovery, the hydrolysis by β-glucuronidase aryl-sulfatase was monitored using genistin (EXTRASYNTHESE™, 1325 S, Genay, France), a pure compound used as a control reagent, in an external standard run in parallel to each measured sample. The compound was dissolved at 1 mg/mL in DMSO as the stock solution. In these operating conditions, the hydrolysis performance was always between 87 and 103%. Recoveries greater than 100% can be explained by the high accuracy of ELISA measurements and inter- and intra-assay variations.

##### Assay

GEN and DAI were assayed in soy matter and treatment water using an ELISA specific to each molecule, as explained by Shinkaruk et al. [37]. The primary antibodies were selected and obtained in previous works [38,39].

##### Characteristics of the ELISA

The soy matter samples were always diluted to 1/50 (*v*/*v*) for enzymatic digestion. The sensitivity of the GEN assay in food matter was 10 µg/mg. Similarly, sensitivity for DAI was 6.5 µg/mg. For water samples, the sensitivity was 100 ng/mL and 65 ng/mL for GEN and DAI, respectively. Based on multiple tests, the intra-assay variation was never over 7%, and the inter-assay variation obtained on different microtitration plates was always below 17% [38,39]. Assays were not considered if the r of the sigmoid calibration curve was <98.5%. The final dilutions in the microtitration plates ranged from 1/50 to 1/800 for soy matter and 1/5 to 1/3200 for water samples. Each sample was assayed in triplicate, using three extracts and on three different microtitration plates. All values in this paper are given in aglycone equivalent.

#### 2.4.2. Folin–Ciocalteu Assay

The phenolic compounds were assayed by the spectrophotometric Folin–Ciocalteu assay, within the wavelength range 750–765 nm [40]. The soy samples were treated to obtain the hydrophobic components. They were extracted twice for 2 h at 90 °C under stirring (magnetic stirrer, Ika Labortetechnik^TM^, Staufen, Germany) with water:ethanol and then methanol:hydrochloric acid mixtures at 50:50 by volume. All resulting samples were then dried in a vacuum oven (Oven Memmert^TM^, Schwabach, Germany) to obtain a dry matter reference which was weighed and then diluted back in water to obtain a final absorbance ranging between 0.5 and 1. The treatment water samples were centrifugated for 10 min at 10,000 RPM (Centrifuge Rotanta 460R^TM^, Hettich^®^, Berlin, Germany) prior to assays. A standard curve was worked out using gallic acid (Sigma, 842649) at concentrations starting from 0 up to 250 µg/mL. For the measurements, 1000 μL of Folin–Ciocalteu reagent 1/10 (Supelco^®^, 109,001, Saint-Quentin-Fallavier, France) was added to 200 μL of either diluted soy matter extracts, water samples or standards. Each mixture was subsequently vortexed and then left to stand for 5 min away from light at room temperature, before adding 800 μL of Na_2_CO_3_ (75 g/L; Supelco^®^, 106,392, Saint-Quentin-Fallavier, France). All mixtures were shaken before being left to stand for 90 min away from light and at room temperature. Then, they were read at 760 nm with a spectrophotometer (Jenway^TM^ 6305, Stone London, UK). The negative control did not contain phenolic compounds and was used as a 0 μg/mL sample to generate the standard curve.

### 2.5. Granulometric Testing Preparation

To assess the dependence of the IF removal process on the beans granulometric factor, dehulled beans were pounded in a cutter (Stephan, ProEx^TM^, Hameln, Germany) to generate different particle sizes. The particles were sieved on a RETSCH^TM^ (Eragny, France) AS200 sieve and subsequently weighed, before being separated into two batches which were washed for 5 min at 25 °C under magnetic or orbital table stirring at 200 RPM. The washing step in ultrapure water was performed at small scale in Erlen Meyer vials. The samples were kept frozen at −18 °C until they were required for further analysis.

### 2.6. Treatment Water Analysis and Processing

#### 2.6.1. Quality Characterisation

Water samples were collected after the drenching-and-washing pilot process for IF analysis. Other parameters, namely dry matter, suspended matter (glass fiber membranes AP 40, Millipore, France)—according to NF EN 872—and turbidity (turbidimeter TL2360 Hach, Düsseldorf, Germany)—according to NF EN ISO 7027-1—were also measured. Particle size distributions were evaluated using laser diffraction (Cilas PSA 1190L Anton Paar^TM^, Orleans, France) according to NF ISO 13320-1, with or without ultrasonic treatments.

#### 2.6.2. Cleaning

Several tests were performed to elaborate the best operative protocol. First, samples of 200 mL each were subjected to centrifugation at 3000, 6000 and 9000 g for 1 min at 20 °C using a lab centrifuge (Hettich^TM^, Tuttlingen, Germany). Based on the results obtained with the spin tests, 3.4 L of this water was centrifuged at 3000 g for 1 min, for solid–liquid separation, in order to maintain a material balance between the suspended matter and the polyphenols, among which are the IFs.

Another cleaning operation was then tested on 1 m^3^ of the same water batch involving a 10-inch-high cartridge filter (PRECart PP 2, 499A110W071SP, PALL, New York, NY, USA) with a filtration area and a cutting of 0.5 m^2^ and 1 μm, respectively. The pressure was fixed at a maximum of 5 bars, and the initial flow rate was set up at 300 kg/h/m^2^. Cartridges were used in a BECO INTEGRA CART 1 carter (EATON Begerow^®^ Product Line, Paris, France). The samples were characterized before and after the filtration operation.

#### 2.6.3. Isoflavones Trapping and Recovery

Previous studies already showed the ability of synthetic resins to fix IFs [41]. Therefore, after cleaning of the pilot treatment water by PRECart PP 2 cartridge filtration, specific adsorption resins were tested for IF trapping and for water recycling.

As a pre-selection step, a jar test was performed, wherein 100 mL of water was exposed to 20 mg of resin under stirring for 2 h, before IFs were assayed. Ten different materials were tested to allow a preliminary selection: Lewatit^®^ VPOC-1064 (Lanxess, Cologne, Germany), AD3008 (Jacobi, Paris, France), PAD900 (Jacobi, Paris, France), PAD950 (Jacobi, Paris, France), SEPLITE LXA838 (Sun Resin, Shaanxi, China), SEPLITE LXA94 (Sun Resin, Shaanxi, China), Relite EXA 90 (Resindion, Binasco, Italy) and Optipore™ SD-2, Amberlite™ XAD7HP, Amberlite™ FPX66, the last three supplied by Dupont^®^ (Paris, France).

The two most promising resins were selected for open circuit column tests to determine the maximum volume of water that can be treated by the resin before performance loss (that is, the achievement of saturation), and to define the IFs’ recovery conditions. For this final selection, 212 mL of the pre-selected resin was pre-wetted, then inserted into a 3 cm diameter column and gradually saturated with 4.2 L of water, representing 20 times the bed volume of the resin. Five samples were collected, each after passing 850 mL of filtrate through the column. The desorption function was checked using either demineralized water or ethanol, or mixtures of both in different volume proportions: 70:30, 50:50 and 30:70.

### 2.7. Statistical Treatments

For the laboratory scale testing, a Tagushi experimental design was chosen, as it allowed the evaluation of numerous variables with relatively few runs [34,35]. The statistical analyses relied on the measurement of signal-to-noise ratios with XLSTAT software (Addinsoft_2020; XLSTAT, Paris, France).

For pilot-scale experiments, each concentration corresponds to a mean value, and its standard deviation is derived from at least triplicate measurements. The significance was obtained by assessing the ν value according to a non-parametric Mann–Whitney test.

## 3. Results

### 3.1. Laboratory Scale

The results of the statistical analysis at laboratory scale can be found in Figure S1 in the Supplementary Materials, which exposes the five factors tested, and their values. It can be seen that only two factors, the temperature and the matrix form, seem to influence mainly the IF removal rate. As for the matter:water volume ratio, contact time and stirring speed, their variations did not result in significant differences in the IF removal rate (*p* > 0.05). The latter appears to be optimal at room temperature and for the smallest particles. With regard to the effect of the nature of soy matter, the data indicate that pounded dehulled beans give the best IF removal result, followed by soy cake (formed from crushed beans) and finally dehulled beans. However, the first two were not retained, because firstly, pounding was not seen as a manageable solution at pilot or industrial scale, and secondly, the treatment water in the case of soy cake was highly turbid, indicating that a large quantity of soluble matter had leaked into it.

**Figure 1:**
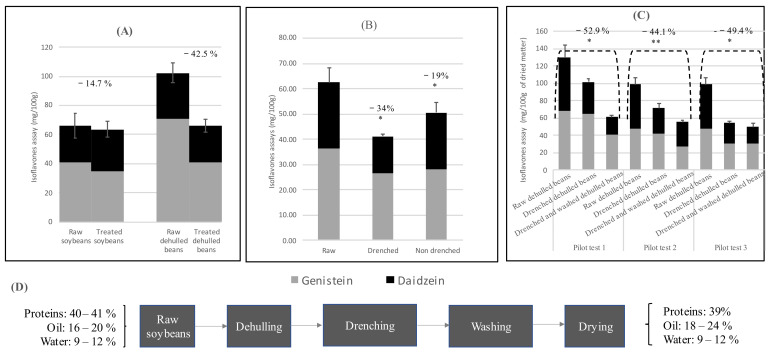
Results of isoflavone removal at laboratory and pilot scales. (**A**) Laboratory-scale average isoflavone removal from raw and dehulled soybeans. (**B**) Pilot-scale mean isoflavone removal from dehulled beans, with and without drenching before washing. (**C**) Pilot-scale mean isoflavone removal after application of the improved process on three pilot trials. Percentages correspond to the isoflavone reductions. Error bars are SEM. Significant differences for a risk α of 5% (*) and 1% (**) are also indicated. (**D**) Full improved pilot process overview with input and output matter characteristics.

As shown in Figure 1A, at laboratory scale, the mean IF removal from raw soybeans was 14.7 ± 5.23%, while it was 42.5 ± 7.56% for dehulled beans. This effect was obtained regardless of the other experimental conditions, in line with the statistical Taguchi analysis (Figure S1), from which the type of soy matter was identified as one main influencing factor. Indeed, it was not possible to significantly reduce IF content in whole soybeans, while up to 60% of the initial GEN could be removed by working on dehulled beans at room temperature for 10 min. It appeared that the beans’ envelope could limit extraction of IFs.

### 3.2. Pilot Scale

As established by the laboratory investigation, the following were retained for pilot-scale operations: dehulled soybeans as a starting material, and room temperature, since no heating was required. The matter:water volume ratio had to be fixed at 1:4 due to a technical limitation leading to deficient homogenization for the least-diluted mixtures, knowing that the 1:5 case did not bring any improvement. The water/soy contact time was also fixed at 10 min, since 5 min was not sufficient to obtain the expected results. As for the stirring speed, it had to comply with technical constraints.

Besides, during the first transposition of the trial to pilot scale, the washing water was turbid due to dust generated by the dehulling step, and the IF elimination efficiency was less than expected based on laboratory tests. This is the reason that a drenching step was added before washing. Furthermore, the dehulling process was shown to generate heterogenous particle sizes. To check the influence of the particle size on the IF removal efficiency, a comprehensive analysis was performed.

Additionally, it should be noted here that drying is a mandatory step in the production of soy-based matter when water pre-treatment is applied, and consequently, it is crucial to restrict the corresponding allotted time as much as possible. Soy-based materials cannot be extruded or sold to a food manufacturer if their moisture levels are higher than 12%.

#### 3.2.1. Effect of Drenching

Figure 1B shows the difference between IF removal rates from dehulled beans that were either drenched or not, prior to washing. Indeed, the soy lost almost twice as many IFs (40.1 ± 7.23 mg/L instead of 25.8 ± 2.54 mg/L) if submitted to the additional step. The differences were significant for total IFs, GEN and DAI at 0.5%, 0.1% and 0.05%, respectively.

Figure 1C shows the result of the pilot trial performed three times. It can be seen that IFs were successfully reduced by 52.9, 44.1 and 49.4% (mean 48.8 ± 4.43%), respectively. The efficiency was better for DAI. The differences in IF removal between the three pilot batches are non-significant, thus validating the process of IF removal at pilot scale.

#### 3.2.2. Effect of Granulometry

**Figure 2:**
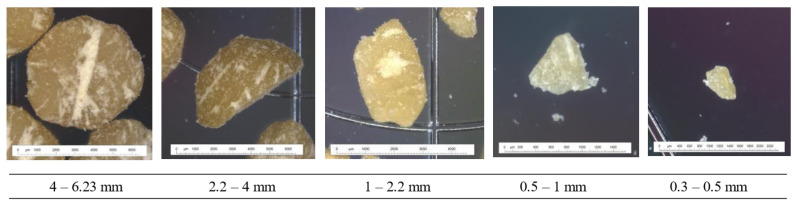
Granulometry of the fractions obtained by sieve analysis on dehulled beans with 2D photographs.

The industrial process of dehulling generated different types of particles. Their sorting by size gave the following proportions in number: 11.53 ± 1.09% whole beans and 88.47 ± 1.16% half-beans. When the proportion was given by mass, whole beans represented 14.35 ± 2.50%, dehulled beans 72.25 ± 3.44%, seed fragments 13.32 ± 4.92% and envelope residues 0.09%. The morphological analysis of the particle sizes generated by dehulling showed solid densities of 1.2–1.4 and 1.2–1.8 whether pounded or not, respectively. It was highest for the fractions 1–2.24 mm and 0.5–1 mm for pounded and unpounded beans, respectively. The details of the particle size sorting are given in Figure 2, with a 2D photograph for each fraction. It can be seen that the particles have an ellipsoidal shape which is more pronounced as the size decreases. Pounding made the particles’ shapes more irregular. The particle sizes generated have dimensions either over 4 mm, between 4 and 2.24 mm, or between 2.24 and 0.5 mm. The 4–6.23 mm fraction corresponds to the most represented mass, i.e., 94% of the total mass of particles recovered on the sieves.

With regard to the impacts of granulometry on the process, the following correlations were observed. The washing effectiveness on deflavonoidisation was slightly better with magnetic than orbital stirring, but the difference was only significant with the smallest particle sizes. The IF content decrease between raw and washed matter was significant, regardless of the particle size. It was also noted that the smaller the particle size, the more efficient the deflavonoidisation, independent of the hydration of the samples, since the values were calculated according to the dry matter (see Figure S2 in the Supplementary Materials). However, in return, the drenching waters were all the more contaminated by solids, the smaller the particle size. This indicates a loss of water-soluble substances that are also present in soybeans, i.e., proteins, sugars, fibres, etc.

#### 3.2.3. Drying of Treated Dehulled Soybeans

**Figure 3:**
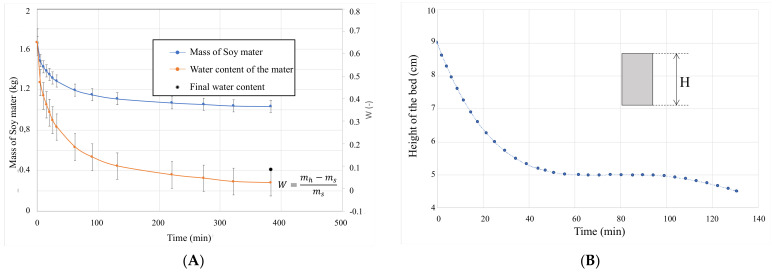
(**A**) Water and mass evolutions during the thick bed-drying process. (**B**) Evolution of the bed height during drying. Operating conditions: air temperature 55 °C; air velocity 1.5 m/s; initial water activity (Wini) 65.0% and final water activity (Wfin) 8.3%.

The required drying time ranged from 1 to 2.5 h, depending on the technique tested and the operating conditions. Thick bed-drying was more effective than lick-drying. The main results obtained on the thick bed are gathered in Figure 3, and also in Figure S5 in the Supplementary Materials. In this case, the mandatory 12% moisture could be achieved in about 1 h. Drenching and washing caused swelling of the dehulled beans by water which was then reabsorbed (shrinkage) upon drying, causing the pile volume to decrease sharply (Figure 3B).

#### 3.2.4. Nutritional Quality

A sketch of the improved processing steps for IF removal at pilot scale is presented on Figure 1D. It can be seen that the protein, oil and water contents of dehulled beans were equivalent before and after being subject to the whole treatment. The protein content went from 40 to 39%. The oil proportion increased from 16–20% to 18–24% (with inter-batch variation), most probably because the removal of the envelope essentially reduced the seeds’ carbohydrate content [42]. Additionally, the targeted water content of 12% was successfully achieved after the drying process, as described above.

In short, the whole process, including drenching and washing dehulled beans with water (20 °C, 10 min, 1:4 matter:water volume ratio and 200 RPM) and then drying them for 1 hour, from now on referred to as the improved process, succeeded in maintaining an important part of the nutritional quality of soybeans, especially the protein and lipid contents.

### 3.3. Comparison of ELISA and Folin–Ciocalteu Method

**Figure 4:**
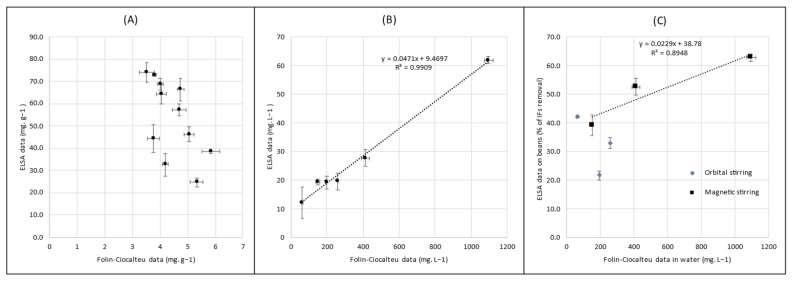
Comparisons between the ELISA and the Folin–Ciocalteu methods for isoflavone content determination: (**A**) in soybeans, a lack of correlation; (**B**) in water, the presence of correlation; (**C**) between IF removal from soybeans (ELISA) and polyphenols in water (Folin–Ciocalteu), the presence of correlation. Error bars are SEM.

Figure 4A represents the correlation between IF concentrations obtained from soybean extracts by ELISA and the phenolic compound concentrations measured by the Folin–Ciocalteu method. It can be seen that the data points are completely dispersed, without any apparent correlation. In fact, it is impossible to quantify the reduction of IFs in soy matter using the Folin–Ciocalteu method. On the contrary, in Figure 4B, which represents the ELISA results plotted against the Folin–Ciocalteu data obtained from treatment water samples, the correlation is strong (R = 0.995). Interestingly, and confirming this result, the correlation between the decrease rate of the total IFs in dehulled beans determined by the ELISA and the polyphenol content in treatment water determined by the Folin–Ciocalteu method is significant, as shown in Figure 4C. Additionally, it is noted that using magnetic stirring, the R correlation coefficient is 0.946, while as mentioned previously, data obtained using orbital stirring are inconsistent.

### 3.4. Treatment-Water Analysis

The mixture made of the drenching and washing waters of dehulled beans contained 2158 mg/L of suspended matter with a turbidity of 4195 FNU (Formazin Nephelometric Unit). Laser diffraction analysis highlighted particle sizes ranging from 0.1 µm to 300 µm [d_10_: 6.35 ± 2.40 µm; d_50_: 106 ± 8.91 µm; d_90_: 192 ± 24.1 µm; d_max_: 400 ± 71 µm.], considering that the few large particles present masked the smallest ones, and most agglomerates were dissociable under mechanical stress. The results of the first centrifugations (200 mL samples) are presented in Figure S3 in the Supplementary Materials. The different suspended matter concentrations obtained at different speeds ranged from 309 mg/L to 217 mg/L in the supernatant. Therefore, the centrifugation step allowed the clarification of the aqueous phase. The pellets obtained after the second centrifugation (3.4 L sample) had a volume of 60 mL. The pellets contained soybean fragments, and therefore proteins and other insoluble soy particles. The percentage of IFs in the dried extract was found to be 13.02 ± 2.27%.

The quality of the drenching and washing water mixture was significantly improved by membrane filtration on a PRECart PP 2 cartridge. This test was performed on raw water without centrifugation pre-treatment. The initial suspended matter concentration was 2158 mg/L and was reduced by a factor of 100 to reach a residual value of 21 ± 11 mg/L after cartridge filtration. Considering the water sample tested, the cartridge filtration, for which the maximum pressure was set on 5 bars, started at a flow rate of 300 kg/h. The filtration flow was decreased progressively to 18 kg/h after 33 min. The amount of water that was filtrated during this period was 22.22 kg. Two filtrates were collected, one (filtrate 1) before pressure stabilization at 5 bars and the other (filtrate 2) when the pressure was kept at its maximum (Figure S4A in Supplementary Materials). The flow was maintained at a constant level of 300 kg/h for 6 min. A mass balance based on measurements performed before and after filtration was carried out for IF concentrations. The data seen in Figure S4B show that 83.33% of all IFs were trapped on the cartridge filter, representing 83.42% for DAI and 82.56% for GEN, specifically. In total, the 22.22 L of treatment water left 45.8 mg of IFs on the cartridge. In parallel, the suspended matter was reduced from 2158 mg/L to 25 mg/L, leaving a mass of 47,300 mg of solids on the filter. Considering these figures, the cartridge extract contained only 0.01% of all IFs.

### 3.5. Isoflavones Recovery

The pre-selection results, as described in Section 2.6.3, highlighted evidence of polyphenol retentions ranging from 30% for acrylic resins to 40–60% for conventional styrenic resins, the performance depending on the pores’ volumes, diameters, and specific surface areas, as shown in Figure S6 in the Supplementary Materials. Remarkable performances were obtained with functionalized resins recently developed by DUPONT^TM^ and SUN RESIN^TM^, with polyphenol reductions over 95%. The various resins tested were all effective in trapping IFs; some of them, such as VPOC resins 1064, Relite EXA 90 and Optipore SD-2, made it possible to lower the water’s residual IF content below the ELISA’s limits of quantification. They correspond to resins with pore diameters between 5 and 10 nm and a styrene-divinylbenzene (DVB) base. Unlike the first two references mentioned, the Optipore SD-2 resin also fixed almost all polyphenols, probably because of its functionalization with an amine group.

Consequently, the choice of the resin to be tested at pilot scale went naturally towards the one most effective for trapping organic compounds present in water, which was Optipore resin SD-2. However, it was likely that the adsorbed compounds would be difficult to desorb due to its functionalization. Thus, a second resin had to be selected in order to satisfy to both the most effective trapping and an adequate desorption. The candidate resin which appeared to fulfil these two constraints simultaneously was Amberlite^®^ FPX 66. It is a non-grafted resin of the same base (styrene-DVB), from the same supplier, which ended up being very effective in trapping IFs (98.7%), even if it trapped only 50% of the total polyphenols.

**Figure 5:**
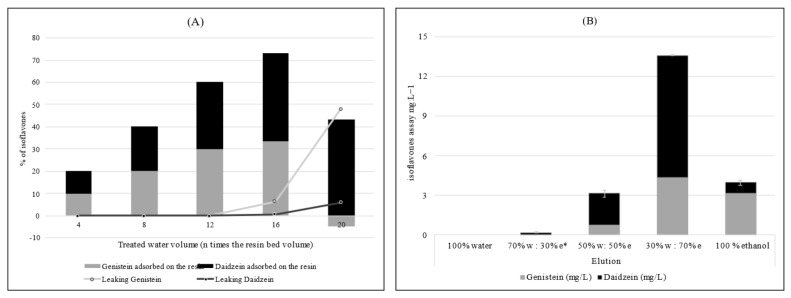
Isoflavones adsorption (**A**) and desorption (**B**) from Amberlite FPX 66^®^ resin. The curves (**A**) represent progressive isoflavones leaking from the resin. * w and e refer to water and ethanol, respectively.

All polyphenols were recovered from Amberlite^®^ FPX 66 resin by a 50:50 water:ethanol mixture, whereas even with 100% ethanol, only 40% were recovered from the Optipore^TM^ SD-2 resin. Concerning the total IFs, the trend is the same and it can be noted that GEN was less readily eluted than DAI, as shown in Figure 5B. A 70:30 water:ethanol mixture did not have sufficient eluting strength in both cases, and a minimum of 50% and 100% alcohol was required for Amberlite^®^ FPX-66 and Optipore^TM^ SD-2 resins, respectively.

Tests on Optipore^TM^ SD-2 resin showed that the polyphenol content of the first filtrate was low (4 mg/L), which confirms the IF-trapping yields obtained in the jar test. The exhaustion of the resin was gradual and only partial, since after filtration of 32 times the bed volume, the suspended matter was only 17 mg/L, which corresponds to a 62% reduction in total polyphenols. The Optipore^TM^ SD-2 resin fixed 223 mg of polyphenols, i.e., a fixing capacity of 1.05 mg/g, without reaching its maximum saturation capacity. For the IFs (44% GEN, 56% DAI), a quantity of 5.73 mg (i.e., 0.027 mg/g) was fixed without reaching saturation.

Regarding the Amberlite^®^ FPX66 resin, during the filtration of 20 bed volumes, the polyphenol content fluctuated between 20 and 25 mg/L, and the reduction yields for polyphenols ranged between 44 and 56%, in line with the results of the jar test. In total, the Amberlite^®^ FPX 66 resin fixed 112 mg of polyphenol, i.e., a fixing capacity twice as low (0.52 mg/g) as Optipore^TM^ SD-2 resin, without reaching its maximum saturation capacity. For IFs, the assay results showed that the Amberlite^®^ FPX 66 resin was less selective, since resin exhaustion appeared after passage of, at most, 16 bed volumes for DAI and GEN. The maximum saturation capacity of the Amberlite^®^ FPX 66 resin was therefore reached for IFs at 2.865 mg (i.e., 0.01 mg/g), which represented 73% of remaining IFs in the treatment water.

## 4. Discussion

### 4.1. Isoflavone Removal from Soybeans

Previous experiments have shown that a 60 min precooking of pounded dehulled soybeans at 90 °C successfully removed 54.32 ± 12.35% of the initial IFs [27]. The present process succeeded in proving that a treatment of only 10 min at 20 °C was sufficient, after drenching, to reach similar IF removal rates (48.8 ± 4.43%) from dehulled soybeans.

The drenching step of dehulled beans was added at pilot scale for cleaning the seeds before deflavonoidisation, as the dehulling and light grinding favoured the appearance of a superficial layer of fat, which trapped a large part of the dust and flour. The latter two, containing IFs, had to be eliminated before washing in order to improve the contact between water and the matrix surface, helping to achieve a better deflavonoidisation. Indeed, both GEN and DAI are mainly present in a glycosidic conjugated water-soluble form (logP < 1) in soybean seeds [43]. This finding was confirmed by IFs’ assay in water. Drenching helped maintain a good concentration gradient between dehulled beans and water, and improved the effectiveness of IF leaching.

Surprisingly, IF removal was better at 20 °C than at higher temperatures. This could be partly explained by the significant decrease in soybean solid content when the temperature rises from 30 to 40 °C [44]. The hypothesis is that proteins and carbohydrate leakage could prevent IF removal. Another explanation may be that cooking at high temperatures could precipitate the most external proteins of the dehulled beans. Such precipitation could reduce the IFs’ transfer to water. This has not been reported in the literature beforehand, thus additional experiments are needed to help understand this result.

### 4.2. Influence of Granulometry

The goal of these measurements was to show the influence of the seeds/the water exchange surface on IFs’ extraction from dehulled beans. Indeed, the data indicate that the smaller the particle size, the larger the specific exchange surface and the greater the IF removal. However, this was true only for the magnetic stirring case. The difference observed between the two mixing techniques on the efficiency of IF removal could be explained by considering that the orbital shaking at 200 RPM was not able to ensure good contact between water and soy matter. Indeed, it is the orbital shaking that generated matter areas that remained dry in the testing bottle. Consequently, the IF measurements were not consistent when compared to those obtained using magnetic stirring.

However, this better IF removal from smaller particles was counterbalanced by a higher leakage of nutritive elements into the water (high turbidity with the smallest particles). Therefore, dehulled beans were considered a better starting material.

### 4.3. Comparison of ELISA and Folin–Ciocalteu Methods

As already mentioned, comparing the ELISA and the Folin–Ciocalteu assay makes sense given the time involved, including the extraction steps, in each case. Thus, after sample collection, the latter technique gives a result in a few hours, while the ELISA requires at least three whole days and one night.

The results of the correlation study showed that the Folin–Ciocalteu method was not able to assess the reduction of IFs in soy matter. The reason is undoubtedly linked to the presence of many insoluble polyphenols in soy products, and to the fact that the amount of water fixed on the material changes with the particle size (more water fixed on the smaller particles). The main discrepancy observed between the two methods is based on differences in their specificity. Indeed, the ELISA used in this study targets specifically GEN or DAI [38,39] and the extraction process triggers the water-soluble fraction, whereas the Folin–Ciocalteu method recognizes all polyphenols based on the chemical reaction of phenol rings with the assay reagents. The extraction performed prior to the analysis also includes non-water-soluble compounds. However, soybeans contain many phenolic compounds, some of which are soluble in water, some of which are not. Indeed, insoluble tannins, saponins, and also proteins, amino-acids, sugars and vitamins, among other food components, were reported to be detected with a Folin–Ciocalteu assay [45]. Thus, it was not really surprising to observe strong discrepancies between the two assay techniques.

On the contrary, the very good correlation between the ELISA and Folin–Ciocalteu results on water analysis was crucial, as it confirmed that the Folin–Ciocalteu method, by measuring water-soluble phenolic compounds, allowed an indirect evaluation of the total IF content in the treatment water samples. As the IF content in these waters is correlated with that in the corresponding treated soybeans, the Folin–Ciocalteu method seems adequate to simply and quickly evaluate the effectiveness of the IF reduction process.

Interestingly, and confirming this result, the correlation between the reduced concentration of IFs in dehulled beans found by ELISA and the amounts of polyphenols in treatment waters determined by the Folin–Ciocalteu assays was significant. A plausible explanation could be that after centrifugation, the water only contains soluble polyphenols, and so potential interference is reduced. Consequently, the Folin–Ciocalteu method could be advantageously used to quickly compare the efficiency of water treatments of different processes designed for IF removal.

It is important to acknowledge here that there is also a sensitivity difference between the two methods. The ELISA’s sensitivity is 1 ng per well, and the detection limit is 0.48 ng per well. The 50% competition points vary from 8 to 50 ng/mL, depending on the assays. The Folin–Ciocalteu standard curve linearity lies between 35 and 250 µg/mL of gallic acid. The detection limit is 0.863 µg/mL, and the sensitivity is 0.0073 OD/µg/mL. These data are given in equivalent gallic acid.

### 4.4. Isoflavones Collection

The resin open-circuit column test with Amberlite^®^ FPX66 allowed the trapping of almost 80% of the remaining IFs in pre-filtered treatment water. However, considering the number of IFs that could be present in general in treatment waters, the fixation capacity of the resin might not be sufficient alone.

Luckily, IFs and other flavonoids are known to adsorb to proteins in their native or glycosidic forms [46]. Therefore, it was not surprising to find them in the pellets after centrifugation. The percentages of IFs in the dried extract (13.02 ± 2.27%) rank within currently commercialized IF extracts that can be found on the pharmaceutical market (2, 10, 20 or 40%). The recovery of dried IF extracts still needs to be tested at an industrial scale, as it can represent a significant quantity. This was not the case for the extract obtained by cartridge filtration, as it contained only 0.01% of IFs. The difference between the two separation techniques (centrifugation and cartridge filtration) comes from their respective cutting rates. With cutting at 1 µm diameter, the filtration cartridge retained more matter than the centrifugation step. In addition, the affinity of the IFs for the PRECart PP 2 cartridge may be low, favouring the water solubility of the glycosylated IFs.

Thus, to obtain the richest possible IF extract, it might be advantageous to combine the pellets produced by the centrifugation with the IFs recovered from the resin treatment of the supernatant. This alternative route should potentially lead to full recovery of IFs from treatment water.

### 4.5. Safety Limits for Isoflavones

The US National Toxicology Program demonstrated in two multigenerational reproductive and carcinogenesis studies that exposure to GEN caused mammary and pituitary gland tumours in female rats, and reproductive impairments in both sexes [31,32]. The efficient daily dietary exposure was 35 mg/kg body weight, thereby defining the preliminary toxic levels in animals. It has been admitted in classical toxicology that in order to translate a minimal toxic dose of a substance from animals to humans, it has to be divided by the product of various safety factors that range from 1 to 10 [47,48,49]. In the case of GEN, three safety factors must be considered. The first is a factor of extrapolation from a LOAEL (lowest observable adverse effect level, which is available for GEN) to NOAEL (no observable adverse effect level). The second is a factor of extrapolation between animals and humans. The third is a factor counting for sensitivity differences among humans. If the commonly admitted values are applied, this brings out a total of 300, even if 184 could be safe enough, as argued by Kodell & Gaylor [48]. Regarding soybeans, the NTP assessed only GEN toxicity, thus the question regarding other estrogenic IFs remains unanswered. Because soybean also contains DAI that can be metabolised into a more potent estrogen, i.e., equal [50], it would be safer, in the authors opinion, to consider the toxicity of total IFs. Since no data are available on DAI toxicity, it seems adequate to apply the highest safety factor to total IF measurements, i.e., 300. Such a decision will bring the IF limit for an adult weighing 60 kg to 20 mg/day, and for a 20 kg child to 6.7 mg/day.

In previous works, multiple soy-based foods from France were analysed for their GEN and DAI contents [25,26]. Several of them, including roasted soybeans, soy milk, soy galettes, and soy cheese or tofu, could bring up to 50 mg or more per portion. Such amounts clearly justify the work presented here. In this study, we managed to reduce dehulled beans’ IF levels from 90–100 mg/100 g to 40–52 mg/100 g. Dehulled beans are usually texturized in soy proteins. If it is assumed that texturization does not change the IF content of textured proteins, the latter could be further treated to achieve acceptable IF levels in final foodstuffs, if required. Luckily, this is starting to be the case in France, and at an industrial scale. Repeated water rinsing of textured soy proteins instead of soybeans can reduce their IF content by up to 90%. One plausible explanation for this high IF removal efficiency could be the protein conformational and molecular changes that occur during texturization, as the protein water absorption has been found to increase with denaturation, due to the formation of a protein matrix that is stabilized by hydrophobic interactions [51]. This water treatment of textured proteins was a preliminary result obtained during the implementation of a new industrial recipe, but it failed to reach the RefD. The implementation of drenching and washing of dehulled beans, on the one hand, and rinsing of textured proteins, on the other, would give rise to highly safe soy-based food that may be eaten without any restriction.

## 5. Conclusions

The present work reports mainly on the study of pilot processes tested to remove IFs from soybeans intended for the preparation of processed foods. The entirely developed process was found to be not only effective in reducing the IFs in soy without decreasing its nutritional benefits, but also water and energy efficient. Indeed, it mainly involves water rinsing and drenching of dehulled beans at room temperature. In addition, as the water used for these treatments should be cleaned up, it was shown that collecting IF-enriched extracts for pharmaceutical applications through cost-effective and easy-to-implement techniques is possible. The results obtained should contribute to the evolution of regulations on the toxicological values of estrogenic IFs. Consumers will thus benefit from the best nutritional values of soy without the potential undesirable effects. However, to fully reach this aim, the improved process developed here must be further tested in association with other water treatment steps on textured soy proteins. All these results remain to be validated at industrial scale.

## Data Availability

The data presented in this study are available on request from the corresponding authors. The data are not publicly available due to confidentiality.

## Supplementary Materials

The following supporting information can be downloaded at: https://www.mdpi.com/article/10.3390/foods12071540/s1. The following supporting information are openly available in supplementary material. Figure S1. Results obtained on tests performed at laboratory scale following the Tagushi L25 experimental design. Figure S2. Test of isoflavone removal by water washing. Figure S3. Water-cleaning operation by centrifugation. Figure S4. Effect of the cartridge filtration on isoflavones’ quantity in treatment water. Figure S5. Characterisation of the thick bed-drying process. Figure S6. Isoflavone measurements in water samples incubated for two hours with different resin types.
