# Supplementary material for: Improved Food-Processing Techniques to Reduce Isoflavones in Soy-Based Foodstuffs
Source: Foods. 2023 Apr 5;12(7):1540. doi: 10.3390/foods12071540 (PMC10093994; doi:10.3390/foods12071540)
Supplement: Supplementary file 1 [file foods-12-01540-s001.zip › foods-2208775-supplementary.pdf]

Supplementary Materials

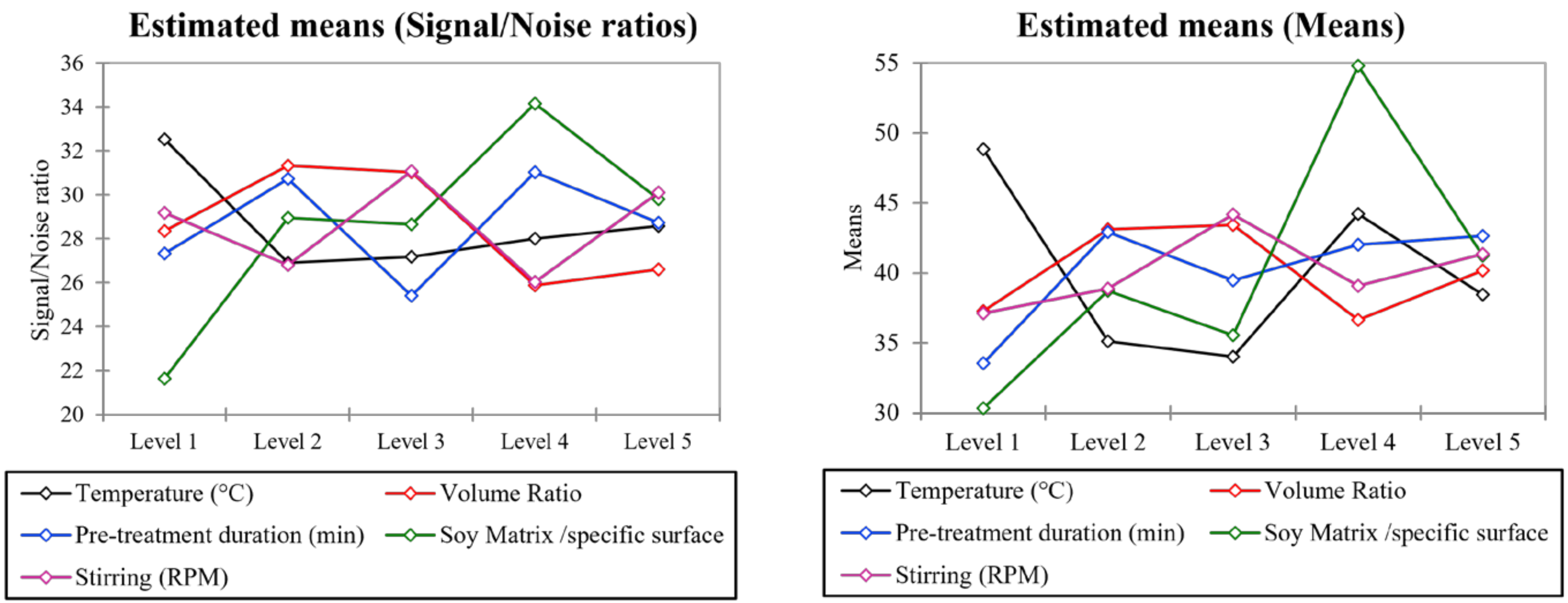

	Level 1	Level 2	Level 3	Level 4	Level 5
Temperature (°C)	23	45	55	65	75
Volume Ratio	1:2	1:2,5	1:3	1:4	1:5
Pre-treatment duration (min)	0	5	10	20	30
Soy Matrix /specific surface	Beans	Dehulled beans	Flakes	Pounded dehulled beans	Soy cake
Stirring (RPM)	0	50	100	150	200

Figure S1. Results obtained at laboratory scale following the Tagushi L25 experimental design. **A.** shows the estimated means taking into account the signal/noise ratios. **B.** shows the means for the different parameters examined. Two parameters exhibit a major effect on isoflavones removal: temperature and soy-matrix i.e. specific surface. The best removal is obtained at room temperature and for pounded dehulled beans. The influence of treatment duration, volume ratio and stirring speed seem to be less crucial.

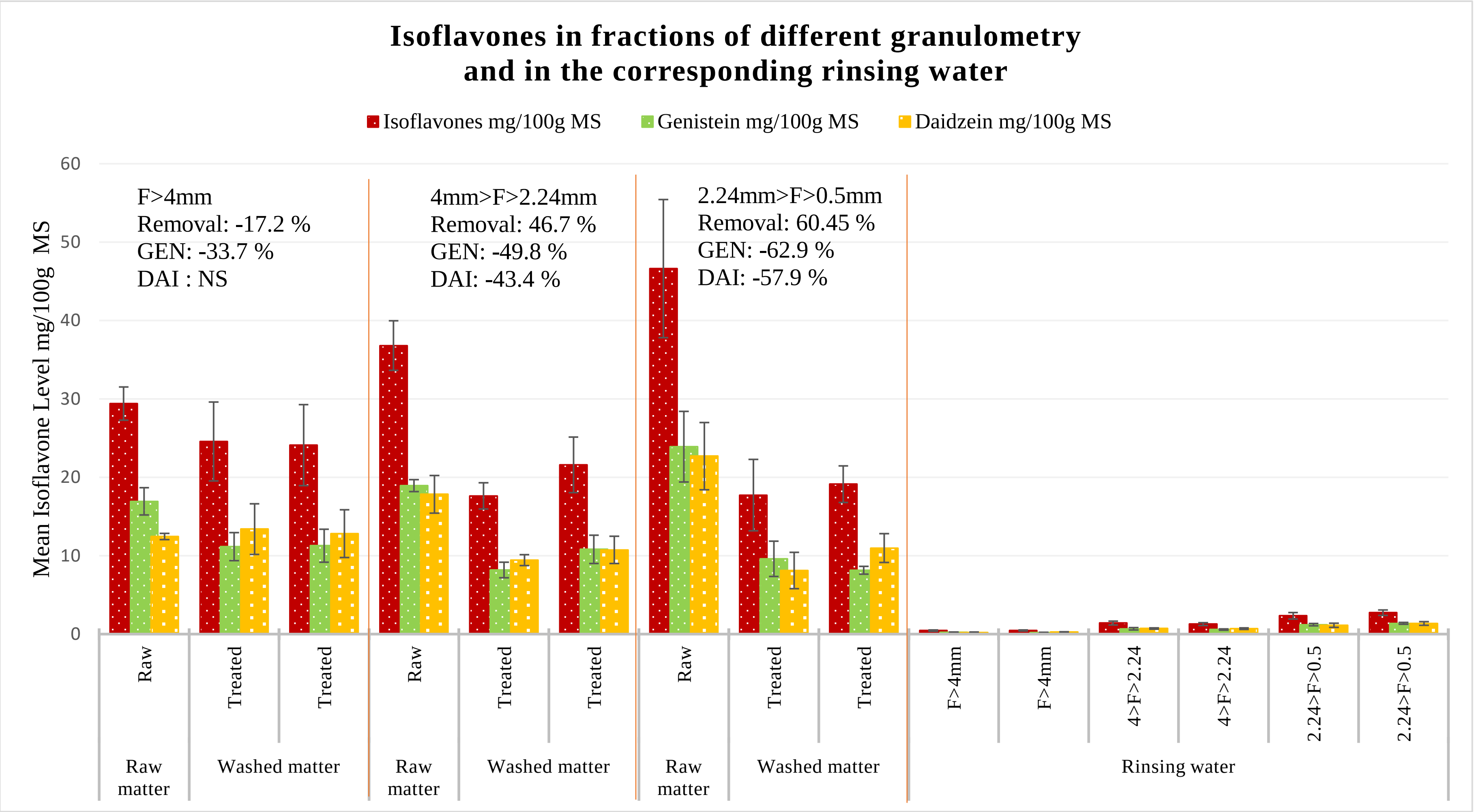

Figure S2. Test of isoflavones removal by water washing. The test is performed for 5 min, at 25°C, in 4 volumes of water, on soybean fractions of different sizes. Fraction 1: F > 4mm; Fraction 2: 4mm>F>2.24; Fraction 3: 2.24>F>0.5. The percentage of isoflavones removal is given for each fraction. In these conditions, daidzein is less efficiently removed than genistein. The removal efficiency is correlated with the isoflavone measurements in the rinsing water. The isoflavone removal efficiency is greater for smaller particles.

Supplementary data 3. Results from the water-cleaning operation using centrifugation

A

**Operating conditions**				
Trial n°	1-1D	2-1D	3-1D	4-1D
Acceleration (G)	3000	6000	9000	9000
Time (min)	1	1	1	15
Loaded mass (g)	202.8	201.8	203.9	202.5
**Supernatant**				
Mass (%)	99.1	99.3	99.0	99.1
Suspension mater (mg/L)	309	271	227	171
Turbidity (NTU)	976	863	730	398
d10 (µm)*	5.69 (1.34)	5.40 (2.34)	3.35 (0.39)	-
d50 (µm)*	116 (4.38)	129 (7.54)	84.5 (6.09)	-
d90 (µm)*	119 (11.5)	232 (214)	195 (203)	-
**Pellet**				
Mass (%)	0.9	0.7	1	0.9
Siccity (%)	17.7	15.8	20.7	22.7

B

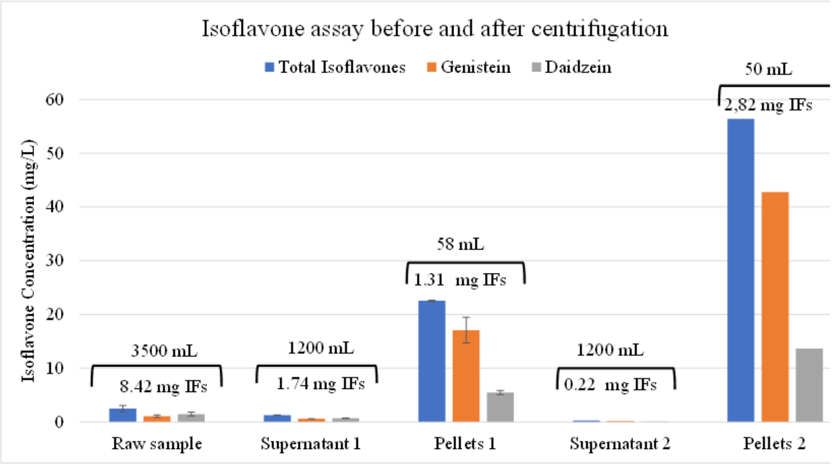

Figure S3. Water-cleaning operation by centrifugation. **A**. Conditions and results of the preliminary tests of centrifugation on the mixture of drenching- and washing-water. **B.** Measurements in the washing-water treated by centrifugation. 3500 mL of washing-water was centrifuged in two batches of about 1000 mL each. Based on the isoflavones measurements in the raw sample, the supernatant and the pellets, the latter appeared to contain between 48% of the initial isoflavones. 15% were trapped in pellet 1 and 33% in pellet 2.

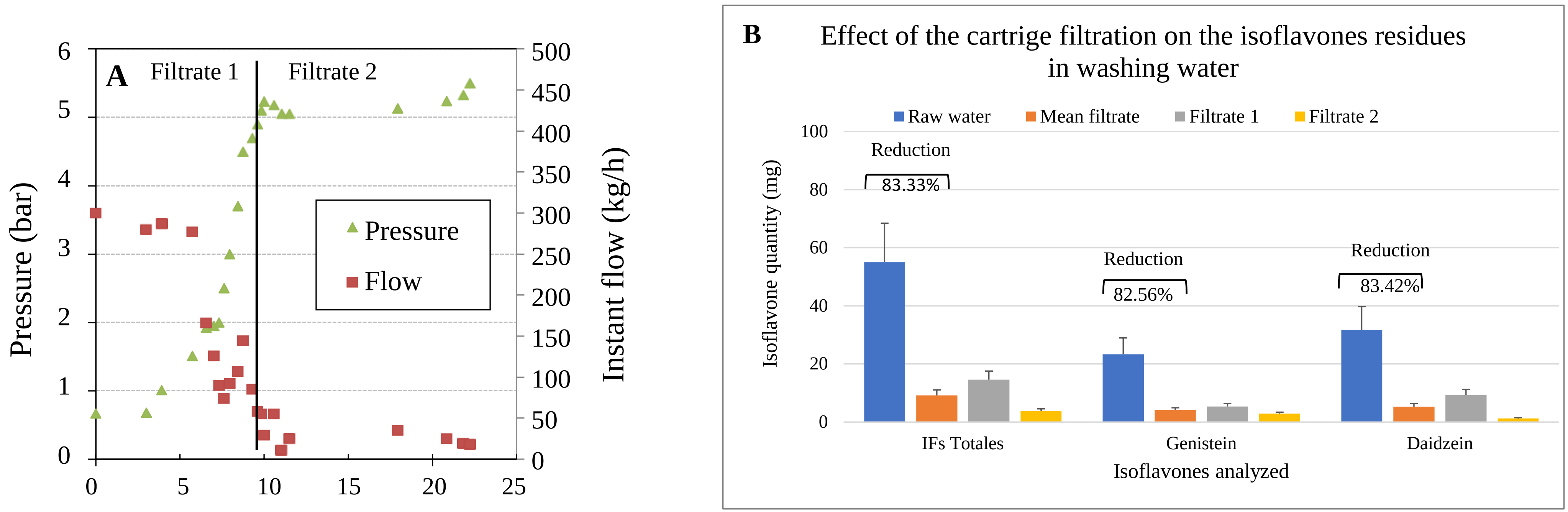

Figure S4. Effect of the cartridge filtration on isoflavones quantity in washing-water. **A.** Evolution of pressure and water flow with the mass of the mixture of drenching- and washing-water filtrated on the PRECart PP2 cartridge (cutting: 1 µm). Two filtrates were collected one before pressure stabilization the other when pressure was stabilized at 5 bars. **B.** Effect of cartridge filtration on the isoflavones residues in washing water. The mass balance allows to determine that about 83% of the isoflavones were trapped on the cartridge.

A. Temperature evolution during drying

B. Evolution or relative moistures at entrance and exit

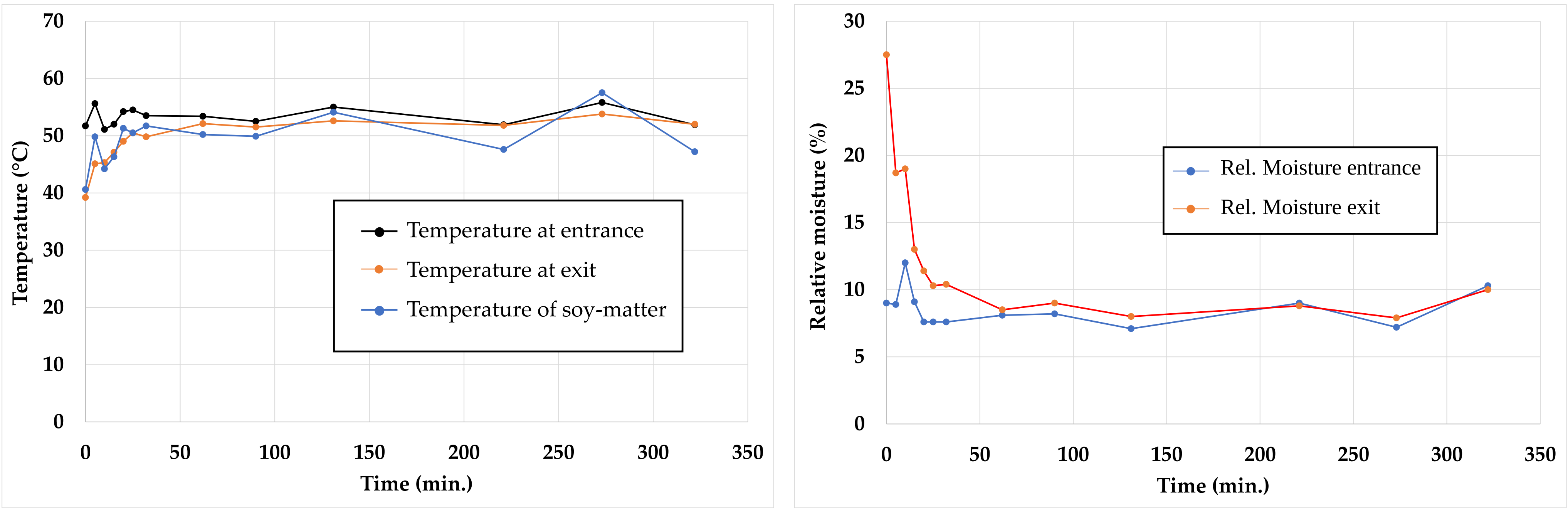

Figure S5. Characterisation of the drying process on thick bed. The wet soy mater (1.665 kg) reaches dryness after 62 min of air treatment. The parameters are: air temperature =55°C; air velocity =1.5 m/s; initial water activity (W) =65,0%; final W=8,3%. **A.** Evolution of the temperature at entrance and at the exit of the thick bed. **B.** Evolution of the air moisture at entrance and exit of the thick bed. The time required to reach the moisture degree required, *i.e.* 12% is around 30 min, in these conditions.

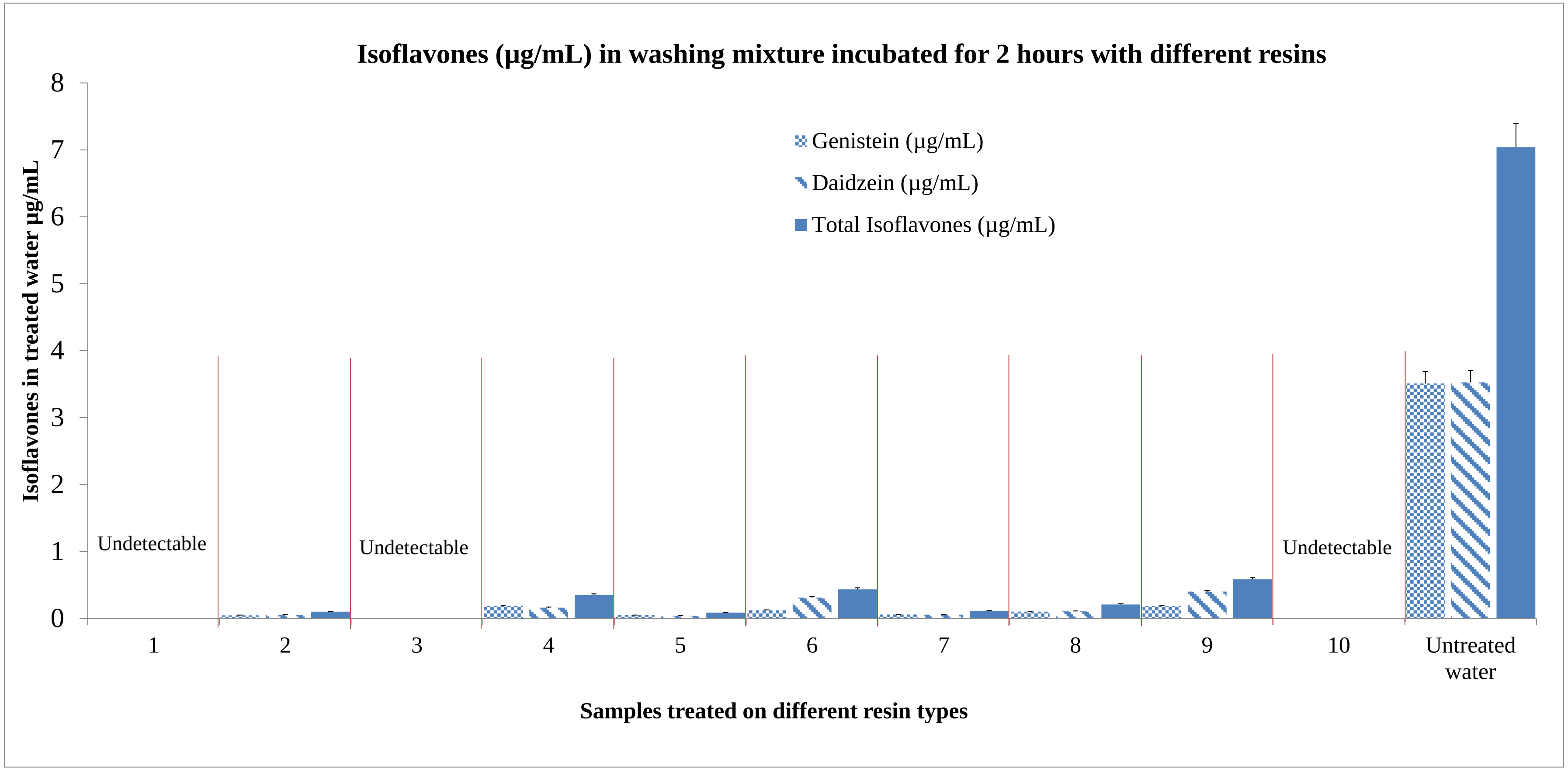

Figure S6. Isoflavone measurements in water samples incubated for two hours with different resin types. 1: Lewatit® VPOC 1064; 2: AD 3008; 3: Optipore™ SD-2; 4: Amberlite™ XAD7HP; 5: ™ FPX 66; 6: PAD 950; 7: PAD 900; 8: SEPLITE LXA 838; 9: SEPLITE LXA 94; 10: Relite EXA 90.
